# Moving Medicine, Moving Minds: Helping Developing Countries Overcome Barriers to Outsourcing Health Commodity Distribution to Boost Supply Chain Performance and Strengthen Health Systems

**DOI:** 10.9745/GHSP-D-16-00130

**Published:** 2016-09-28

**Authors:** Priya Agrawal, Iain Barton, Roberto Dal Bianco, Dana Hovig, David Sarley, Prashant Yadav

**Affiliations:** a Merck & Co., Inc., Kenilworth, NJ, USA. Merck & Co., Inc. is known as MSD outside the United States and Canada; b Imperial Health Sciences, Gauteng, South Africa; c The Bill & Melinda Gates Foundation, Seattle, WA, USA; d University of Michigan, Ann Arbor, MI, USA

## Abstract

Senegal and other developing countries are improving access to health commodities by outsourcing supply chain logistics to private providers. To achieve broader, lasting reform, we must support further adoption of the outsourced model; assist country-led cost-benefit analyses; and help governments build capacity to manage contracts and overcome other barriers.

## INTRODUCTION

Until recently, women in Senegal who went to their local public health clinics seeking a means to prevent pregnancy had about a 3 or 4 in 5 chance of leaving empty-handed.^1^ Implants, pills, injectables, and other contraceptives, while promoted as part of Senegal’s commitment to improving maternal health and access to family planning, were routinely out of stock. The problem, all too common in developing nations, was improper supply chain management, specifically inventory management and delivery. To address this, the government of Senegal embarked on a major overhaul of its supply chain for 9 different contraceptives (with 2 more added later). Within 6 months, stock-outs ceased almost entirely.^1^^,^^2^

Key in the success of Senegal’s reform was the government’s strong commitment to, and effective implementation of, hiring private third-party logistics providers (3PLs) to manage orders and handle deliveries from district warehouses to local health facilities, with clear benefits for service levels and costs. One analysis compared cost and service through the use of private operators versus those of government employees performing the same activity in a different region of the country. The analysis found that outsourcing decreased the proportion of facilities experiencing stock-outs from over 80% to less than 2%^1^ while reducing distribution costs by 36% annually.^3^

By outsourcing supply chain logistics to private operators, Senegal decreased the proportion of facilities experiencing stock-outs from over 80% to less than 2%.

These gains were achieved during a 3-year program supported by MSD for Mothers (known as Merck for Mothers in the United States and Canada), The Bill & Melinda Gates Foundation, IntraHealth International, and other partners. During the program, other components developed by the private sector were implemented, including an innovative inventory management system and new methods of data tracking to support mobile warehousing. These made stock replenishment more responsive to real consumption. Contracted workers are able to accurately track and verify consumption and stock levels and plan and customize shipments; regular data uploads allow them to quickly address potential issues.

Qualitative analysis and cost comparisons between regions being served by 3PLs and the one region that was not served by 3PLs helped garner support within the government for the outsourced model. In summer 2016, the new system began transitioning from donor-supported partners to government control under Senegal’s National Supply Pharmacy (PNA), a process that is expected to take several months. At the launch of the handover process in August, the Director of the PNA pledged to continue contracting with private operators for last-mile deliveries to all 1,400 of the country’s service delivery points, and to expand the system to cover approximately 100 products by the end of 2017. Senegal’s Minister of Health and Social Action has endorsed the outsourced model as a way to help ensure access to essential medicines and health commodities for all citizens. This political support is both encouraging and necessary, as long-term success will depend on the government’s ability to institutionalize the approach.

A handful of other developing countries are also pursuing outsourcing and reaping the benefits. In the Western Cape Province of South Africa, for example, the storage, handling, and transportation of vaccines has markedly improved in the hands of a 3PL while costs have also been lowered.^4^ Kenya outsources the transport of medical supplies to all its health care facilities, having determined that managing a fleet of trucks was neither a core competency of government nor an effective use of government resources.^5^ In Nigeria, direct deliveries from an outsourced provider have increased the availability of vaccines in Kano and Lagos,^6^ and, despite constraints from the economic downturn and weaknesses in management capacity, the direct delivery model is gradually being expanded nationally. In Mozambique and Zimbabwe, direct deliveries of a range of products have increased stock availability, enhancing system performance.^7^^,^^8^ In other places, however, such as Togo, similar programs have not been as successful. The success of direct-delivery programs managed by the private sector ultimately depends on strong government commitment; clear roles and accountability mechanisms; a robust technical design of the processes and key performance indicators to be measured; and effective multi-stakeholder collaboration.

Success of direct-delivery programs managed by the private sector depends on strong government commitment, clear roles and accountability mechanisms, robust technical design, and effective multi-stakeholder collaboration.

We urge other developing nations—in Africa and elsewhere where public-sector supply chains have been underperforming for years—to look to these countries’ experiences as examples of what can be achieved and reminders of the issues that will need to be addressed (Table).

**TABLE:** Outsourcing Supply Chain Logistics in Low- and Middle-Income Countries: The Advantages, the Hurdles, and Some Recommendations on How to Move Forward

Advantages	Hurdles	Recommendations
Outsourcing saves money: Using private operators to distribute medicines and other health commodities can reduce costs by as much as one-third or more.^a^	There is general reluctance by in-country leadership to give up government ownership of delivery systems and infrastructure.	Evaluate what lies within government’s core competency, and what lies outside of it. Through oversight and enforcement of strict service-level agreements, governments can maintain control over their supply chains even while outsourcing the logistics; government still owns and manages the process, even when non-government drives the trucks.
Built-in performance incentives: Logistics operators are motivated to provide effective, efficient service and prevent stock-outs in order to win and keep their contracts.	There is insufficient capacity and expertise within governments to write and effectively manage contracts with private operators.	Assist governments with building effective contract management and enforcement teams.
Medical staff at service delivery points are no longer responsible for restocking inventory and can focus on patient care; new jobs are created in the local private sector as local companies hire more employees and otherwise invest in their own growth to secure government contracts.	Outsourcing the logistics of health commodity distribution could threaten to eliminate some public-sector jobs.	Governments may need help restructuring their human resources and retraining and reassigning employees for oversight and supervisory roles; local private operators may need training and other support.
In a government-run distribution system, employee working hours and staffing levels are fixed; private operators can be more flexible, so government pays only for what it needs and uses.	In some markets, it can be difficult to find private-sector operators trained and equipped to use state-of-the-art approaches to inventory management and to comply with strict requirements regarding storage and transport of medicines.	Private-sector donors or businesses, acting as fourth-party providers, can assist in the professional development of local private-sector operators.
Contracting with multiple operators can help spread risk, so that the failure of one is far less likely to bring a whole system down.	Concerns exist about long-term financing and sustainability of the new health distribution system given the temporary nature of donor support.	Private-sector expertise can help with the necessary costing analysis, but top-level, in-country leadership must take ownership over the process—and the solutions.

a Outsourcing in Senegal reduced distribution costs by an estimated 36%,^3^ and outsourcing the distribution of vaccines in South Africa yielded significant savings, from 28% of the vaccine cost to 6%.^9^

## THE ADVANTAGES

When deciding whether to outsource, there are many benefits to consider. For example:

- Outsourcing improves performance through competition. It brings market forces into play, with competitive bidding and performance-based incentives. To stay competitive, 3PLs invest to acquire the technical expertise and capital assets they need to provide top service while keeping prices in line. Top performers earn contracts. If they fail to perform, another company gets the job. Poor performance by public-sector personnel is more difficult to rectify.
- Private operators are highly incentivized to comply with the strict storage requirements governing health commodities. Those who fail to do so risk termination of their contracts. (For these incentives to work, of course, governments must be willing to hold those service providers accountable.)
- Private 3PLs are better-equipped to manage growth. Government agencies typically lack the flexibility and capacity to scale quickly to address seasonal issues or outbreaks, due to rigid budgets and bureaucratic hiring processes.
- 3PLs typically add stronger information and data management systems, enabling greater stock management and resource efficiency. (Data, and data visibility, are central to supply chain performance.)

Outsourcing improves performance through competition.

For all these reasons and more, outsourcing is standard practice across Canada, Europe, and the United States, and in the rest of the developed world. In fact, no country in the Organisation for Economic Co-operation and Development (OECD) has a government-run public health supply chain.^10^^-^^12^ The U.S. Centers for Disease Control, for example, has achieved significant cost efficiencies outsourcing storage and distribution for its multibillion dollar Vaccines for Children program^13^; the UK National Health Service, which outsources procurement, storage, and distribution to DHL, has projected a savings of a billion pounds over 10 years.^14^^,^^15^

Developing countries stand to benefit from outsourcing in two other critical ways. First, with private contractors handling deliveries and other on-the-ground logistics, doctors, nurses, and other clinical staff can concentrate on their primary responsibility and core competency: patient care. In a government-run system, it is the medical personnel, already in short supply across the developing world, who are often saddled with part-time supply chain duty, filling out inventory forms, traveling to storage sheds to collect stock, and performing other tasks for which they were not originally trained. When outsourced, skilled 3PL workers make the deliveries and perform the inventory control. This task shifting is a key benefit of Senegal’s outsourced distribution model. It has allowed government employees to be redeployed to new clinical support and management positions, not downsized, as is often feared.

Outsourcing logistics operations to private contractors frees up doctors, nurses, and other clinical staff to concentrate on patient care.

Second, government spending through outsourcing stimulates the local economy by creating business opportunities for local entrepreneurs that in turn create new private-sector jobs. In Senegal, local operator Cabit SA has doubled its staff from 30 to 60 since being contracted by IntraHealth International to handle logistics. In Malawi, contraceptives and some essential medicines (including antimalarial drugs) are distributed by Cargo Management Logistics (CML), a local private company that has grown from 46 to 112 employees in the 4 years since taking on this work. In Nigeria, Imperial Health Sciences manages warehousing services and deliveries by 7 locally owned and financed transport companies, which together employ more than 270 people to deliver products to 5,000 points of care.

Outsourcing stimulates the local economy by creating business opportunities for local entrepreneurs.

## THE CHALLENGES

Given all the upside, why has outsourcing failed to catch on in the developing world? Here, we provide a few explanations. First, outsourcing requires a sharp break with an old tradition of moving medicine through government-owned centralized stores. This colonial practice was later reinforced when Ghana gained independence and tried to emulate the supply chain model used in the former Soviet Union. The Soviets owned and operated the medicine supply chain, so Ghana and many other countries in Africa did the same. However, this model has long proved inefficient and fragile, hobbled by weak performance incentives and the inability to flex capacity as needed. By the 1990s, the model was already struggling across Africa and it buckled when the HIV crisis hit. Subsequent efforts, with international assistance, to sustain this model have done little to alter the fundamental structure, which continues to prove incapable of handling the ever-widening scale and diversity of products required to meet patient needs.

**Figure:**
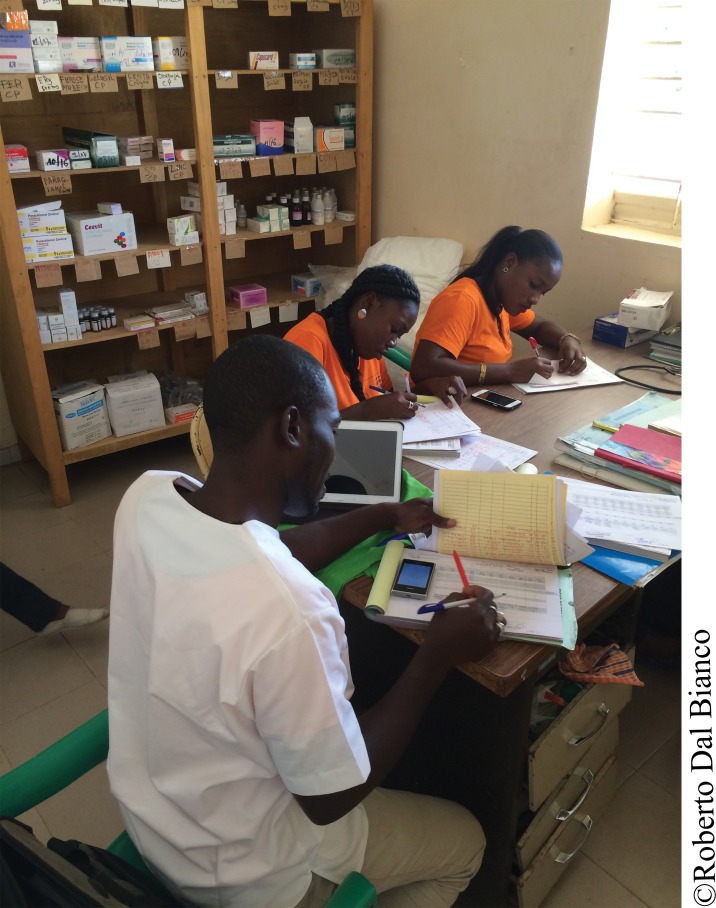
Employees of Cabit, a 3PL hired to handle last‐mile distribution of contraceptives and other health commodities, record consumption and delivery data in a stock room of a health center in the Fatick region of Senegal.

Second, many developing nations lack the mechanisms and capacities for contract management and oversight. And it can be difficult to find local private operators capable of handling health product distribution. In some countries, private logistics operators may only serve urban areas. For example, Beale et al.^16^ and VillageReach^17^ highlight the lack of transport providers in rural Mozambique. Private companies require long-term contracts to invest in transport capacity in an underserved region where they do not currently serve a market. Yet governments are hesitant to make long-term contracts as for many this is not yet a proven model. For transporters, especially smaller local players, the timeliness of government payment is also critical for their cash flow.

Many developing nations lack the mechanisms and capacities for contract management and oversight.

Third, concerns that outsourcing threatens public health sector jobs can dampen political support. Nobody wants the prospect of laying people off, particularly in countries where the public sector is the primary source of stable employment.

In addition, while outsourcing can reduce product diversion and pilferage, a robust, transparent, and rigid tender process for the outsourced contract award is a prerequisite for its success.

Finally, there is the question of sustainability. Donor-funded programs are often the way that outsourcing is introduced and tested in a country. Yet it is often the pattern that such efforts prove initially successful, only to revert to insourcing when the program transitions back to government. Kenya, for example, reverted back to insourcing some of its distribution activities when an outsourcing project supported by the U.S. Agency for International Development (USAID) ended. Concerns about long-term financing—and the temporary nature of donor support—have made many country leaders reluctant to take the necessary steps toward change, even as they readily acknowledge a pressing need for it. The benefits of outsourcing and direct delivery are based on the combination of technical design (who manages inventory control, information flow), source of financing (donor financed vs. domestic financing), and commitment from political leaders or heads of public agencies. The benefits can start declining when one of these ingredients is missing or becomes diluted over time.

## THE WAY FORWARD

To overcome these barriers, we need to support country leaders as they determine which segments of their supply chains are best suited for outsourcing, and as they seek to identify what truly lies within the core competency of government and what lies outside it. They will need support to adequately assess risks, costs, and potential savings, as well as their own abilities to manage contracts with outsourced partners. And they may need help restructuring internal human resources for new supervisory roles within the new system. The 2015 Gavi study, “Outsourcing the Distribution Component of Vaccine and Medicine Supply Chains,” carried out by Transaid,^18^ offers a useful framework for these strategic discussions.

For those governments considering a move toward outsourcing their supply chains and to all supporting partners and stakeholders—public and private—we recommend the following:

- Conduct an in-depth, cost-risk-benefit analysis of the existing government-run, insourced distribution system. Have an honest discussion about what changes are feasible and who is going to pay for it. The private sector knows how to do the kind of granular costing analysis that is required, and does it well.
- Top-level country leadership should drive the process from the beginning, so that government—including the finance ministry—takes ownership of both the analysis and the solutions.
- Take full account of the many hidden costs of a government-run distribution system, such as asset depreciation and staff time spent away from their clinic post. If not exposed, these hidden costs can make an outsourced model, in which costs are more explicit, seem expensive by comparison. Spending more may make sense if it means dramatically improved product availability on shelves.
- Make clear the return on investment.
- Procure 3PL services through a competitive process to keep costs in line.
- Consider having a commercial or NGO partner serve as a fourth-party provider (4PL) to manage subcontracts with several 3PLs—which Mozambique, Nigeria, Senegal, and Zimbabwe all have done. A 4PL adds value by streamlining the government’s role in the nitty-gritty logistics and in performance management, by creating a single point of engagement. The 4PL can find, train, and manage all local transport partners, and it has the incentive to make sure they do well. (In Senegal, IntraHealth International, acting as a 4PL, provided training for Cabit SA and other 3PL employees on the new inventory management techniques adopted as part of Senegal’s newly revamped distribution system.) Engaging a 4PL represents an additional cost layer, however, and can be a disincentive to outsourcing unless the benefits are clearly understood.
- Build in a transition plan from the start to ensure sustainability after donors pull out. The main challenges facing Senegal during its transition will be managing the higher costs and greater data collection and demand planning requirements posed by a tenfold increase in the number of 3PL-distributed products.
- Explore how existing government staff might be retrained and reassigned to new roles in contract management and oversight. In an outsourced system, government is still in control of the public supply chain, so it is government that must enforce service-level agreements and ensure that performance targets are achieved. Look at pharmacists and others who have been pulled away from their field to work the supply chain and allow them instead to return to what they originally studied.
- Develop sufficient government capacity for contract management and other mechanisms for effective private-sector engagement. Rigorous government oversight is essential to secure compliance, prevent corruption, and ensure that drugs are not misused or mishandled. Contracts will also need to include the proper incentives to ensure that 3PLs distribute to rural and remote areas, not just the major towns and other densely populated regions. (USAID’s experience through its DELIVER Project highlights the importance of building a strong contract management team,^19^ and VillageReach’s experience in Mozambique highlights similar challenges.^8^) Key performance indicators—the ability of outsourced partners to manage costs, quality of service, responsiveness, etc.—must be carefully designed and monitored. Strong contract language by itself is not enough; overseers must be willing and able to enforce the terms and conditions.
- Select individuals with prior private-sector logistics and supply chain experience to serve as top-level supply chain managers. Those with a commercial background are more likely to make the bold (and sometimes politically unpopular) decisions necessary to implement change. Progress in Kenya has been widely attributed to the fact that an executive with rich private-sector experience was brought in to run the Kenya Medical Supplies Authority (KEMSA). Under his leadership, KEMSA has become less bureaucratic, more independent, and more competitive and a good model for other countries looking to outsource and otherwise streamline their operations.
- Engage the public in the debate over supply chain reform, by communicating what’s at stake for consumers—in other words, how a well-functioning supply chain helps protect, improve, and save lives. One way to do this is to present performance metrics in terms that are relevant and relatable to patients. Are the medicines and other supplies available when they need them? Are they affordable and accessible? How far do patients have to travel to get to them? Activists and civil society can play an important catalytic role by shining a spotlight on poor performance and advocating for more reliable access. Sweden, the last industrialized nation to embrace private sector-run medicines distribution (in 2009), tapped a bipartisan, independent agency to monitor and review the successes and deficiencies resulting from the privatization. By highlighting issues important to the general public (not just supply chain experts), the agency allowed government to build public confidence in the project.^10^ If we can similarly generate public interest in supply chain issues in developing countries—and engage civic groups and other community organizations, which can often sway the course of public health policies and programs and bring about greater transparency—we can galvanize public support for the solutions.

Top-level country leadership should drive the outsourcing process from the beginning.

Global stakeholders should build a stronger evidence base for where, when, and how outsourcing works in a long-term, sustainable way in developing country health systems. This will create a global public good in terms of knowledge of what any country starting to consider outsourcing should watch out for, and how to remedy failures and create the right prerequisites for success. Before embarking on such projects, global agencies must also pay close attention to the complex political economy surrounding such projects that is present in many low- and middle-income countries. They must develop a clear path to more effectively communicate its benefits and risk mitigation approaches and bring on board government stakeholders who will strongly advocate for this.

## CONCLUSION

Demands on in-country supply chains will only intensify going forward. The sheer volume of drugs and other products flowing into the public health pipeline increases year after year, as new treatment options become available and governing bodies establish more ambitious health targets. The newly adopted Sustainable Development Goals call for universal access to reproductive health care. The new “90-90-90” targets from the Joint United Nations Programme on HIV/AIDS (UNAIDS)—by 2020, 90% of people living with HIV will know their HIV status, 90% of people diagnosed with HIV will receive sustained antiretroviral therapy, and 90% of people receiving antiretroviral therapy will have viral suppression—will essentially double the number of patients who will require treatment over the next 5 years. The need to ensure continuous access to maternal and child health commodities, as well as the rising prevalence of hypertension, diabetes, and other non-communicable diseases in developing countries, will add further strain. Supply chains will have to be even stronger, more efficient, and more effective to enable governments to meet the needs of their citizens. The recent Ebola outbreak is a stark reminder that supply chains must be agile and responsive—and a reminder of the tragic consequences when they are not.

Supply chains will have to be even stronger, more efficient, and more effective in the future to enable governments to meet the needs of their citizens.

We believe that to get public health supply chains—and patient access—to where they need to be, private-sector outsourcing needs to be part of the equation. Health ministries can and should learn from the Senegal experience, and from the successful efforts of other countries to outsource parts of their supply chains. We believe that many of the obstacles are surmountable and that in most cases, with the proper conditions and support, outsourcing can improve health commodity availability and help strengthen health systems.

The women of Senegal are already benefitting from their country’s supply chain overhaul in family planning. Since the reform, a young wife trying to space her pregnancies to avoid complications, the mother of 6 children who is in no condition to have a seventh, and all the other women who require contraceptives, have been able to depend on the availability of contraceptive products in a way that was unheard of just a few years ago. This is what a top-performing supply chain can do, and it is what every person, everywhere, deserves.

## Notes

**Peer Reviewed**

## References

[1] Daff BM Seck C Belkhayat H Sutton P. Informed push distribution of contraceptives in Senegal reduces stockouts and improves quality of family planning services. Glob Health Sci Pract. 2014;2(2):245–252. 10.9745/GHSP-D-13-00171. 25276582 PMC4168620

[2] IntraHealth International. Expanding the Informed Push Model: October progress report (Internal). Chapel Hill (NC): IntraHealth International; 2015.

[3] Dal Bianco R IPM cost-effectiveness of private vs public sector distribution. Presented at: International Conference on Family Planning; 2016 Jan 27; Nusa Dua, Bali.

[4] Lydon P Raubenheimer T Arnot-Krüger M Zaffran M. Outsourcing vaccine logistics to the private sector: the evidence and lessons learned from the Western Cape Province in South-Africa. Vaccine. 2015;33(29):3429–3434. 10.1016/j.vaccine.2015.03.042. 25819709 PMC5357745

[5] Yadav P Kenya Medical Supplies Authority (KEMSA): a case study of the ongoing transition from an ungainly bureaucracy to a competitive and customer focused medical logistics organization. Washington (DC): World Bank; 2012 Available from: http://documents.worldbank.org/curated/en/2012/04/20330086/kenya-medical-supplies-authority-kemsa-case-study-ongoing-transition-ungainly-bureaucracy-competitive-customer-focused-medical-logistics-organization

[6] Department of Logistics and Health Commodities. Review of the vaccines supply chain transformation activities in 2015 and plans for 2016 (Internal). Abuja (Nigeria): National Primary Healthcare Development Agency; 2015.

[7] Rosen JE Bem J Wolf K Evaluation of the Zimbabwe Assisted Pull System (ZAPS): baseline report. Arlington (VA): USAID | DELIVER PROJECT, Task Order 4; 2015 Available from: http://deliver.jsi.com/dlvr_content/resources/allpubs/countryreports/ZWAssiPullBaseRepo.pdf

[8] VillageReach. Mozambique Dedicated Logistics System (DLS) performance report: Cabo Delgado, Gaza, Maputo and Niassa 2015. Seattle (WA): VillageReach; 2015 Available from: http://www.villagereach.org/wp-content/uploads/2016/03/All-Provinces_PerformanceReport_2015_FINAL.pdf

[9] PATH. Outsourcing vaccine supply chain and logistics to the private sector. Seattle (WA): PATH; 2012 Available from: https://www.path.org/publications/files/TS_opt_outsourcing_br.pdf

[10] Yadav P Transition from state monopoly to managed markets for delivering pharmaceutical products in Sweden: a case study in managing markets for health. Washington (DC): World Bank Institute; 2016.

[11] Organisation for Economic Co-operation and Development (OECD). Competition issues in the distribution of pharmaceuticals. Paris: OECD; 2014 Available from: http://www.oecd.org/competition/competition-distribution-pharmaceuticals.htm#docs

[12] Yadav P Health product supply chains in developing countries: diagnosis of the root causes of underperformance and an agenda for reform. Health Systems & Reform. 2015;1(2). 10.4161/23288604.2014.968005 31546312

[13] Vaccines for Children. Vaccines Management Business Improvement Project (Internal). Atlanta (GA): U.S. Centers for Disease Control and Prevention; 2014.

[14] NHS Supply Chain. Customers on track to unlock £150m savings through NHS Supply Chain [Internet]. 2015 9 21 [cited 2016 Mar 15] Available from: https://www.supplychain.nhs.uk/Home/News/Company/On%20Track%20To%20Unlock%20150m%20Savings

[15] DHL Supply Chain. NHS Supply Chain: innovative procurement and logistics model drives substantial savings for UK’s National Health Service. Milton Keynes (UK): DHL Supply Chain; [date unknown] Available from: http://www.dhl.co.uk/content/dam/downloads/uk/Logistics/nhs_dsc_lifescience_case%20study_v2.pdf

[16] Beale J Mashiri M Chakwizira J Prospects for leveraging private sector logistics firms to support rural access to healthcare: some insights from Mozambique. : Proceedings of the 34th Southern African Transport Conference (SATC 2015); 2015 7 6-9; Pretoria, South Africa p. 329.

[17] VillageReach. Evaluation of health system transport capacity and demand: Mozambique case study. Seattle (WA): VillageReach; 2014 Available from: http://www.villagereach.org/wp-content/uploads/2009/08/062014-TSS-Assessment-Report-FINAL.pdf

[18] Transaid. GAVI study -- outsourcing the distribution component of vaccine and medicine supply chain. London: Transaid; 2015 Available from: http://www.transaid.org/knowledge-centre/gavi-study-outsourcing-the-distribution-component-of-vaccine-and-medicine-supply-chains/

[19] USAID | DELIVER PROJECT. Technical brief: logistics outsourcing and contract management in public health settings. Washington (DC): John Snow, Inc; 2014 Available from: http://deliver.jsi.com/dlvr_content/resources/allpubs/logisticsbriefs/LogiOutsContMana.pdf

